# Functional Infrared Thermal Imaging: A Contemporary Tool in Soft Tissue Screening

**DOI:** 10.1038/s41598-020-66397-9

**Published:** 2020-06-09

**Authors:** Stephanos Ioannou

**Affiliations:** Alfaisal University, College of Medicine, Department of Physiological Sciences, Riyadh, 11533 Kingdom of Saudi Arabia

**Keywords:** Diagnostic markers, Predictive markers, Prognostic markers

## Abstract

Soft tissue injury screening faces two main diagnostic challenges. One is the perceptual bias of the athlete in terms of referred pain and second injury assessment tools are not only in need of highly specialized personal but they are also financially demanding. Since ankle sprains is one of the leading soft tissue injuries, the current study was set to examine the suitability of functional infrared thermal imaging (fITI) in evaluating physiological alteration on the ankle as a result of exercise or injury. The current study consists of a case report of a patient with an ankle sprain and the behavior of temperature after a series of physiotherapy sessions. Moreover to strengthen the communication of the report, results from a healthy population sample were added to draw a deeper understanding on physiological temperature manifestations on soft tissue. Twenty participants underwent a 30-minute treadmill run with pictures of their ankles being taken during rest and after exercise. In addition the case of a patient is reported that has suffered an ankle sprain followed for a period of over a month. It was observed that the temperature of the ankles of participants that underwent physical exercise rose on average by 2.4 °C after taking into account both the medial and lateral sides of the both ankles. In addition the patient’s left ankle appeared to be 2.5 °C above the temperature of the non-affected right ankle. This phenomenon of unilateral hyperthermia of the left injured side seems to start to dissipate by the 21^st^ day following the injury, completely resolving by the 42^nd^ day achieving bilateral isothermia in both ankles. Thermal imaging provides a reliable tool for the screening of soft tissue strain and injury. The current study further expands the literature on soft tissue screening with the use of thermal imaging, adding a quantifiable way for assessing the extend of tissue damage. The implemented method of analyses offers a suggested simple way not only in visualizing trauma but also physical strain. Nevertheless further investigations with a variety in the severity of ankle sprains need to be applied in order for thermal imaging to be used as a first line tool for the assessment and recovery of ankle sprains.

## Introduction

Over the years with the progress of technology thermal imaging has managed to make leaps on ergonomics capitalizing on the progress of nanotechnology and lens engineering. Functional infrared imaging in now portable and more sensitive on infrared dynamics compared to what it used to be when the technique first begun its journey with the Pyroscan in 1959 in Bath^1^. As a result of sensitivity and design infrared thermal imaging has now gained a reasonable exposure over the last 20 years specifically in the potential that it holds in a clinical setting specifically for soft tissue screening^2^.

Acute injury presents the organism with two types of challenges; prevent a possible infection as well as repair the wound and heal the body. To facilitate the physical needs of the body a population of different cells is recruited such as platelets, endothelial cells, macrophages, lymphocytes neutrophils and fibroblasts^3^. Heat, swelling, redness and pain usually accompany inflammation, due to factors such as the release of endothelial nitric oxide (NO) and prostaglandins (PGI_2_ and PGE_2_), platelet derived growth factor and endothelial growth factor can lead to elevated heat patterns^3^. Whereas the former can lead to vasodilation, the later can enhance proliferation and angiogenesis. The change in metabolic and vascular distribution from the “norm” makes tissue thermal changes visible on the infrared spectrum^4^. Asymmetries in the circulation of focal body heat above <1 °C are an indication of an abnormality. Inflammation in its majority is measured on subjective scales that rate pain, redness, gross functional measures (movement/resistance) and volumetric changes such as swelling^5^. All of these subjective measures are not quantitative methods and during assessment the clinician also deals with the personal error bias of pain reference.

Overall healthy people exhibit a bilateral symmetry in the distribution of heat^6^ with physical strain increasing mean temperature values^7,8^. Usually cutaneous temperature differences do not exceed 0.25 °C, while those above 0.65 °C are constantly associated with pathology^9^. Ankle injuries of over 200 patients indicated a minor injury that would resolve in 1–2 weeks when both legs showed isothermia (equal temperature) with the surrounding tissue. Unilateral hyperthermias ranged from 1 to 4 °C between the place of injury and the surrounding tissue; when this thermal asymmetry between the sprained ankle and the unaffected leg ranged from 1.5° to 2.0 °C rehabilitation took approximately four weeks^10^. Recent studies have observed that ankle sprains show changes in temperature as a result of injury with temperature variation increasing based on the severity of damage. This increase in temperature was particularly profound when the affected ankle was compared to the non-affected site^11^. Rodriguez-Sanz *et al*., (2017) postulated significant temperature alterations post exercise in elite athletes that have previously suffered from gastrocnemius-soleus ankle equinus post exercise due to injury sensitivity^12^. Inflammatory arthritis is also another instance in which the underlying pathophysiology makes infrared imaging a perfect candidate for not only detecting but also monitoring the healing process of limbs. Sanchez, Lesch, Brammer, Bove, Thiel, & Kilgore (2008) with the use of a handheld thermal camera illustrated that rats experimentally manipulated to develop a paw edema or arthritis had an increase in the temperature of their paws^13^. Moreover the study also showed a reversal in the mean heat pattern of the paws of the rats after a pharmacological intervention was applied. Other scholars in the field agree that thermal imaging has the ability to verify subcutaneous underlying joint pathology^14–16^. Furthermore evidence from sport injuries have shown that elevated heat patterns are seen in occasions of muscle fatigue^17^ as well as tendon insertion problems such as in the occasion of tennis elbow^18^. Currently there is the mainstream view that future studies on thermal imaging should aim at improving our knowledge of muscle contraction particularly on the “association of temperature and muscle projection”^12^ (pp. 8).

Moving from hyperthermia to hypothermia, decreased local perfusion and colder temperatures have been associated with an increase in catecholamine and vascular constriction as an effect of sympathetic or unmyelinated nerve involvement. Dermal temperature decreases can also be an effect of hypersensitization of alpha-receptors as an effect of denervation^19^. Moreover it has been shown that hypothermic asymmetries in ankle injuries that resulted from damage to the tissue and surrounding structures (termed as posttraumatic reflex sympathetic dystrophy or posttraumatic pain syndrome,) can result from efferent vasoconstriction from afferent C-nociception^20^. Long lasting injuries are associated with a hypothermic effects due to reduced muscle contractility, reduced range of motion, reduced metabolic output and in extreme cases muscle atrophy^21^.

Thermal imaging can give a direct feedback on the physique of an athlete whether the user is a medic, a scientist, coaching personnel or even the same athlete^22^. Moreover infrared imaging provides an avenue for assessing the efficacy of treatment in regards to muscle soreness^23^ and further delivers a feedback on muscle fatigue countermeasures^8^. Due to the non-technical and user-friendly nature of infrared methodology this assessment could be performed even by amateurs contrary to clinician, which could also acquire some more advanced knowledge of image segmentation to pinpoint specifically the origin of the ligament stretch or rupture. By using thermal imaging one can gain insight into the type of injury, extent of damage, as well as recovery rate and rehabilitation^24^. The non-invasive nature of the technique as well as the on-sight assessment can save athletes from longer rehabilitation times and further damage since athletes often wait for weeks or even months before they seek help for a sustained injury^6^. Due to perceptual bias athletes cannot pinpoint the direct source of physical pain but the specificity and sensitivity of the thermal imaging can direct the focus of the physician or therapist locally resolving the issue much faster. Regular screening of an athlete before and after an injury can help the coach or trainer adapt the load according to the pressure put on joints, ligaments and muscles thus always monitoring and avoiding injuries of overtraining as well as allowing enough recovery time prior to the next training session.

The current study was designed to explore not only the suitability of thermal imaging as a novel soft tissue-screening tool^2^ but also lay a methodological framework for approaching abnormal and normal temperature patterns. To communicate this research objective the physical strain imposed on the ankles of healthy participants before and after physical exercise has been evaluated by means of infrared light variability. Finally to better delineate the communication of the current findings a case report has been included of a patient with grade II ankle sprain was included followed over a period of recovery.

## Method

### Ethics

Ethical approval for this study was granted by the institutional review board (IRB) of the Prince Faisal Bin Fahad bin Abdulaziz Sports Medicine Hospital (16–002) in accordance with the declaration of Helsinki and the law of research ethics of living creatures of the National Bioethics committee of the Kingdom of Saudi Arabia. Ethical clearance was granted before patient recruitment was initiated. Patients were recruited for this study following informed, written consent, and data obtained did not alter or affect their treatment. Data was collected and stored in a blinded, anonymised manner, and participants could withdraw at any moment upon request.

### Participants

#### Healthy controls

A minimum number of 20 participants took part in the study of which 12 were males and 8 were females between 30 to 33 years old. Participants were recruited based on healthy BMI and no previous history of ankle sprains or injuries. Participants independent of age, gender and cultural background could participate in the study. Exclusion criteria included peripheral neuropathy, arthritis, previous surgery or fracture as well as heat altering agents.

#### Patient with ankle sprain-case series

To examine the suitability of thermal imaging to identify soft tissue damage CARE case report criteria were followed for the communication of the results the patient with the sprained ankle^25^. The patient was a 60 year-old male who has suffered an ankle injury following a game of basketball. Upon admission to the clinic the patient was complaining of severe pain to his left ankle and had difficultly walking. During physical assessment the clinician examined the site of injury by assessing the mobility of the left ankle through a series of provocative maneuvers aiming at grading the severity of the damage^26^. The left ankle compared to the right appeared swollen and red whereas when gently palpated the patient complained of severe pain. The clinician advised for an x-ray to exclude the occurrence of a fracture, which was negative. Based on the physical assessment and the mobility of the ankle the physician concluded that the patient has suffered a grade-two ankle sprain involving the left anterior talofibular ligaments^27^. The patient was prescribed analgesics and non-steroidal anti-inflammatory drugs, an ankle compressive sock as well as four physiotherapy sessions.

### Data extraction & analyses

#### Pre-Post workout healthy controls

Thermal video pre-processing and thermal data extraction was performed using the software Researcher by FLIR (http://www.flir.com). The final data sets were analysed using the Statistical Software Package for the Social Sciences, version 17 (SPSS, Chicago, IL). Infrared pictures were first examined for any thermal anomalies first through image segmentation before and after exercise. Three different heat zones were created for each image starting from the maximum heat pixel obtained on the temperature gauge and going down by 0.5 °C. The gauge was adjusted for all participants from 28.3 to 35.4 °C. Through this method we obtained three different heat zones of interest red (Max), green (Mid) and blue (Lowest) obtaining a broader understanding on the distribution of heat before and after exercise. After the initial thermal screening, temperature was extracted on two different angles bilaterally (laterally and medially) obtained vertically at a 50 cm distance. To perform data extraction a rectangular ROI was formulated (using FLIR TOOLS) which had as a focal point the anterior talofibular ligament where 70% of ankle sprains originate. According to literature it is the weakest link of the lateral ligament complex^28^. The area of the ankle pre and post workout that was digitally marked using a custom made rectangular region of interest never varied in size across images. Both the mean and maximum heat patterns were recorded away from direct areas of ventilation or heat. To rule out potential cardiac artefacts that could relate to increments in the temperature of the ankle the temperature of the wrists was also taken into consideration before and after exercise.

All temperatures entered a paired-sample t-test analyses (with Bonferonni adjustment). Prior of performing the paired sample t-tests and to control for heart rate thermal artefacts that could potentially affect our results the slope of change before and after exercise was calculated on the wrists since compared to the ankles the wrists had no pressure as a result of impact during the treadmill run. This adjustment was based on the maximum obtained pixel of the wrists after exercise and was subtracted from the temperature of ankles (only for the post-workout data set). To obtain a more homogeneous sample the maximum temperature from the ankles before and after exercise was only used for the data analyses to try and control as much as possible for homeostatic effects of perspiration.

#### Patient with an ankle sprain

For data extraction we have followed the same approach as the one mentioned above for healthy controls were the gauge was adjusted and the image was segmented in three different heat zones. The only difference was the fact that temperature was not extracted through a rectangular ROI and the communication of the results was based on the description of the three heat zones over period of 42 days until biological movement was restored through a series of physiotherapy sessions.

Temperature acquired from the ankle sprain was extracted laterally and medially from both ankles bilaterally. Three masks with an incremental threshold a 0.5 °C were again applied as presented in the result section. Nevertheless due to the fact that this was an isolated case of a patient not enough data was collected to conduct a parametric or a non-parametric statistical test. The results presented are just descriptive of temperature behaviour.

### Power analyses

To examine the number of participants required to have enough statistical power to detect a significant effect (*α* = 0.05), a power analyses was conducted using the software G*Power^29,30^. To examine the differences before and after exercise using a paired sample t-test (two-tailed) a minimum sample size of 17 participants will be needed with a large effect size of *d* = 7. This sample size will give an 81% chance of correctly rejecting the null hypotheses of no difference between pre-and-post exercise temperature values of the ankle.

## Procedure

### Pre-Post workout healthy controls

All participants gave their written consent prior of the experimental procedure and all questions regarding the experimental protocol were verbally answered. Prior of any thermal recordings the participants were asked to remove their shoes and socks for approximately 15 minutes for thermal acclimatization purposes. After the images were acquired for the pre-workout condition all participants run on the treadmill for approximately 30 minutes on an intensity of approximately 8–10 km/h. Rodriguez-Sanz *et al*., (2017) illustrated that a 30 minute of soccer practice provides enough strain on the ankle to examine tissue and subcutaneous micro-vascular alterations^12^. After the completion of the exercise the participants were asked again to remove socks and footwear and rest for approximately 15 minutes after which thermal images were acquired on two different angles of both feet (laterally and medially). In the occasion of the patient, images were acquired again from two different angles.

For the thermal data acquisition a digital FLIR T650sc portable infrared camera was used with temperature sensitivity +/− 1o C and a sampling rate of 0.02 s. This camera has the possibility of recording temperature changes of 0.01 °C. To cancel noise effects caused by the sensor’s shifts/oscillations the camera was blackbody calibrated prior of the data acquisition and the camera was adjusted to record at 640 × 480 pixels per image. All recordings took place away from direct heat and ventilation sources. It was a pre-requisite that during screening, no temperature altering agents were consumed.

## Results

### Healthy controls before and after exercise results

All temperature data sets were corrected based on the incremental maximum value that was observed on the wrists and the ankles of the participants after exercise^31^. The reason why the sample was corrected on the maximum slope of change instead on the mean slope of change was due to the fact that the mean values across participants were not consistently showing the same temperature pattern. Occasionally heat patterns were either showing an increase or a decrease possibly because of perspiration thus it was wiser to focus on the maximum incremental pixel values that consistently showed an incremental tendency. Prior of conducting a paired sample t-test all four data (right/left ankle lateral; right/left ankle medial) sets before and after exercise were examined for normal distribution and outliers that could potentially affect the analyses. To examine these assumptions a new variable was created that represented the temperature difference between the pre-test scores at the region of interest (Temperature at rest) with the post-test scores (Temperature after exercise) of the ankles. To create this new variables the treatment phase was deducted from the control. Thus the new four variables of interest was the result of the maximum temperature of the ankle during exercise minus the temperature of the ankle at rest (AnkleMax°C^exercise^ − AnkleMax°C^rest^). The same approach was applied also on the temperature of the wrists. Descriptive statistics showed that for all four data sets no violations of skewness < 0.8 and kurtoses <2 were observed with all values being less than the critical cut-off point. Shapiro-Wilk’s normality tests yielded a non-significant result *p* > 0.05 for all four temperature regions of interest Left Ankle Medial/Lateral and Right Ankle Medial/Lateral. Finally no outliers were observed based on the box plots with no values being listed on the lower or upper whisker. Before conducting pairwise comparisons the maximum temperature of the right and left ankles during exercise was deducted separately from the average maximum temperature change of the wrists *M* = 0.54 °C to eliminate potential heart artifacts (AnkleMax°C^exercise^) − (WristMax°C^exercise^ − WristMax°C^rest^). The average maximum increase in temperature on the right wrist ranged from 0 °C to 1 °C, *M* = 0.56, *SD* = 0.29 whereas for the left wrist from 0.2 °C to 1 °C, *M* = 0.52, *SD* = 0.22. Paired sample t-tests were then conducted to examine the temperature variations that were present on the ankle of each participant before and after exercise (Table 1).

**Table 1:** Paired sample t-tests representing the temperature increase of the left ankle lateral (LAL), left ankle medial (LAM), right ankle lateral (RAL), right ankle medial (RAM) before and after exercise of the healthy sample.

ROI	Mean Pre	SD Pre	Mean Post	SD Post	df	Mean Diff.	T-test	95% Conf.	Sig. *	η^2^
LAL	32.24	1.51	34.8	0.85	18	2.56	9.18	1.97–3.14	0.000	0.82
LAM	32.68	1.20	35.41	0.70	18	2.73	11.42	2.22–3.23	0.000	0.88
RAL	32.85	1.20	34.88	1.26	18	2.03	22.06	1.83–2.23	0.000	0.96
RAM	33.02	1.32	35.34	0.74	18	2.32	10.10	1.84–2.81	0.000	0.85

*p values Bonferroni adjusted to 0.015.

**Figure 1:**
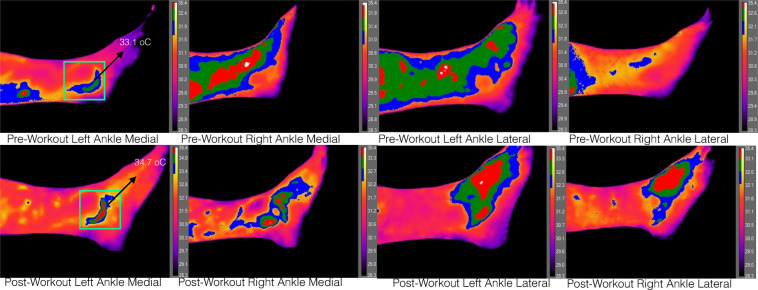
Infrared images before and after exercise indicating the temperature tendency of the population sample.

All tests on the four regions of interest yielded a significant increase in temperature p < 0.015 with a large effect size as proposed by^32^ (pp. 284–7). The average change in temperature after exercise was 2.43 °C (Fig. 1).

Finally an independent sample t-test found no difference in the temperature of the left or right foot during rest and during exercise indicating bilateral isothermia (Table 2).

**Table 2:** Independent sample t-tests comparing temperature changes bilaterally during rest and during exercise of the ankles.

ROI: Pre Workout	Mean Right	SD		Mean Left	SD	df	Mean Diff.	T-test	95% Conf.	Sig. *	η^2^
Medial	33.02	1.32		32.68	1.20	36	0.33	0.81	−0.49–1.16	0.43	0.002
Lateral	32.85	1.21		32.24	1.51	36	0.61	1.37	−0.29–1.51	0.18	0.002
**ROI: Post Workout**											
Medial	35.34	0.74		35.41	0.70	36	0.07	0.29	−0.54–0.41	0.77	0.017
Lateral	34.88	1.26	34.8		0.85	36	0.08	0.24	−0.62–0.79	0.06	0.05

*p values to 0.05.

### Patient with ankle sprain results

**Figure 2:**
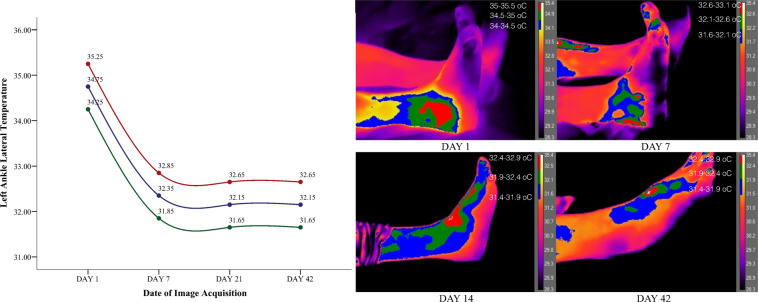
Left lateral injured ankle segmented on three different heat zones representing the tendency of temperature until recovery.

**Figure 3:**
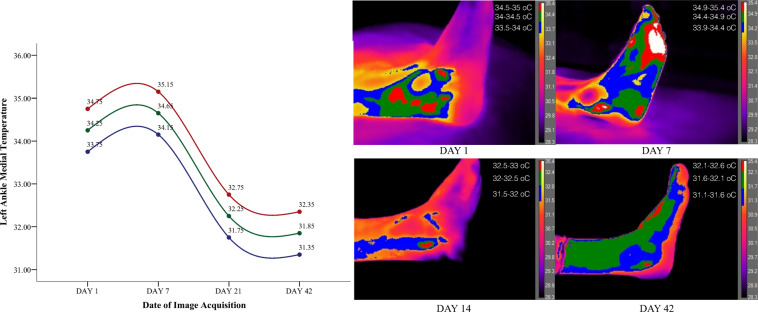
Left medial injured ankle segmented on three different heat zones representing the tendency of temperature until recovery.

**Figure 4:**
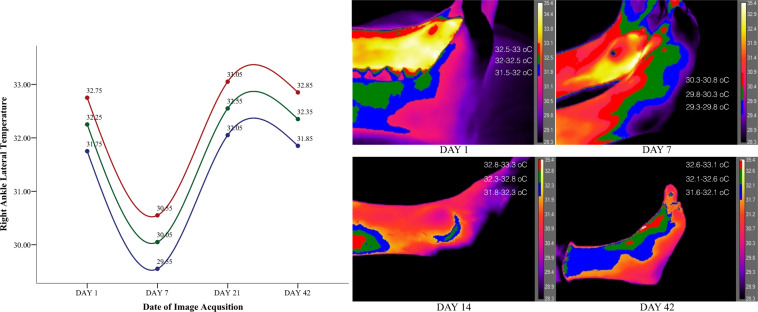
Right lateral non-injured ankle segmented on three different heat zones representing the tendency of temperature up to the recovery of the left ankle.

**Figure 5:**
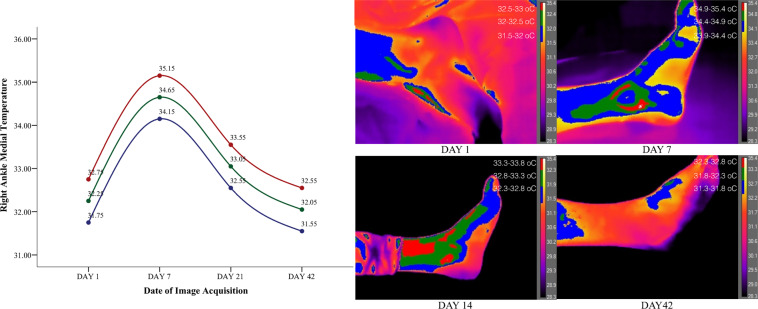
Right medial non-injured ankle segmented on three different heat zones representing the tendency of temperature up to the recovery of the left ankle.

The patient’s symptomatic foot was examined in four separate time periods, at the date of injury, one week after, in 21 days and 42 days following the injury as per^6,10^. Thus in total 16 images were taken from the participant to map the injury and recovery of the ankle. To better describe the temperature tendency all thermal images were segmented in three different colour zones with an incremental value of 0.5 °C ranging 1.5 °C in total where red represented the maximum temperature, green the median, and blue the lower selected temperature values from the top range of the heat gauge. The temperature range (gauge) for all images was set between 28.3–35.4 °C to ensure that all images appear at the same temperature range isolating in this way visually the foot from the background, ensuring visual homogeneity across all collected images. Based on the ankle segmentation and the collected temperature range four different graphs have been created that represent the tendency of the temperature across the four different time periods (Figs. 2–5). The graph represents the average temperature value of each ankle segment based on the temperature range of 0.5 °C that defined each color. Descriptively the injured left ankle shows elevated heat patterns compared to the unaffected right side.

## Discussion

Findings indicate that exposure to a 30 minute run leads to an elevation in temperature of approximately 2 to 2.5 °C. The results obtained represent the maximum temperature change of the ankle after indirectly controlling for an increase in heart rate as well as perspiration resulting from physiological homeostasis. Temperature monitoring suggests that exercise does impose an increment on subcutaneous tissue as a result of physical activity. Moreover temperature comparisons between the left and right foot yielded a non-significant result independent of condition suggestive of a healthy population sample.

Observations on the sprained ankle based on temperature segmentation of the foot point out to a set of clearly defined zones of inflammation with the focal site of injury being defined by the highest temperature values. Heat on the focal point of injury is dispersed even further on the surrounding tissue with a maximum temperature decrease of 0.5 °C. Finally temperature fades away even more with a difference of 1 °C from the maximum obtained temperature (defined in red) creating in this way three potentially suspicious zones of inflammation. Temporally and as early as the 7nth date following a sprain temperature starts to behave differently. The lateral side of the injured left ankle decreases in temperature along with the lateral side of the right healthy ankle by approximately 2 °C. On the 21^st^ date temperature starts moving towards baseline values with the temperature of the injured leg marking a decrease of 0.2 °C from the 7^th^ date following the injury. On the other hand temperature on the healthy ankle increases from the 7^th^ to the 21^st^ day by 2.5 °C. Moreover temperature on the medial side of the injured left ankle marked a minor increase from the 1^st^ to the 7^th^ day of injury of 0.5 °C. In agreement with the injury, the healthy medial right ankle also showed a temperature increase from the 1^st^ to the 7^th^ day of 2.5 °C that dissipated by the 21^st^ day. Overall temperature on both feet started returning to baseline values after the 21^st^ day and both ankles bilaterally showed isothermia by the 42^nd^ day with a maximum difference of 0.30 °C.

Literature postulates that exercise imposes a strain on soft tissue a phenomenon that is reflected in the emission of heat by the muscle^7,8^. Previous research has observed that 1–2 minutes of intense treadmill exercise on elite endurance swimmers and cross-country skiers was enough to lead to temperature elevation on the lower extremities of endurance swimmers but not cross country skiers^22^. Compared to the current findings where an elevation in temperature was observed in all participants following the experimental protocol by^12^ there were certain essential differences with the pilot study of^22^. All participants were subjected to a 30 minute run with all of them being experienced resistance training athletes. So not only the imposed strain was different but also the population sample differed since the participants were not professional athletes. This observation brings in line another potential difference beyond the scope of the chosen experiment. Heat dissipation and homeostatic balance could be affected by the type of regular exercise in which athletes are accustomed to thus subjecting athletes to an unfamiliar physical load could also potentially lead to different heat patterns. The above observation becomes even more apparent when you look into the study of^24^, who have concluded that temperature on trained compared to untrained athletes behaves temporally differently. Under the same conditions of physical strain trained athletes had the ability to raise their skin temperature much faster than untrained participants. On the contrary^33^ did not observe an elevation in temperature but rather a decrease when trained runners were subjected on a 30-minute treadmill run. Nevertheless this conclusion was reached by observing the whole body temperature and not just only the lower extremities. The present study did not reach any significant outcome when temperature of the two feet was investigated for temperature asymmetries. It was observed that during rest temperature of the ankles bilaterally differed by 0.34 °C (medially) and 0.61 °C (laterally). Although a significant result was not obtained literature suggests that asymmetries in temperature above 0.65 °C are indicative of suspicious pathological manifestations^6^. Despite this above-mentioned observation during rest our sample has reached a bilateral isothermia on both ankles after exercise with temperature differences on the feet not exceeding 0.10 °C.

Major temperature asymmetries have been identified during image segmentation between the affected limb and the unaffected site of injury of 2.5 °C both laterally and medially. Findings are in agreement with the observations made by^10^ who postulated that temperature difference between the healthy site and the affected limb can range from 1.5° to 2.0 °C. On the contrary posttraumatic reflex sympathetic dystrophies or posttraumatic pain syndromes manifest in an opposite hypothermic pattern since the governing physiological mechanism is vasoconstriction^20^. Oliveira, *et al*. (2016) argue that there is a temperature decrease when the site of ankle injury is compared to the non-affected ankle. Although the results of^11^ are not in agreement with the current findings, the authors have reliably observed between participants a temperature increase based on the severity of the ankle sprain from grade two to grade three. In extent as the authors postulate participants have received topical administration of cooling agents (e.g. ice packs, ointments) and non-steroidal anti-inflammatory drugs taken as a result of the injury. This is a common practice for the management of acute soft tissue injuries but strongly counter indicative for experimental infrared imaging recordings. Compared to the focal point of injury the surrounding tissue does show a difference. However especially at the beginning of the injury inflammation and vasodilation seems to be dispersed in the surrounding tissue. Previous studies that have compared the site of injury with the surrounding tissue mention a temperature difference of 1 °C to 4 °C^10^. However as we have observed, differences from the focal point and the surrounding tissue are more to the level of decimal places rather than full units, at least based on the severity of the injury of our patient.

Thermal imaging provides a novel tool for the investigation of tissue strain as a result of physical exercise. The technique also provides impressive sensitivity for the spatial identification of injuries, which could help an orthopedic, or a physiotherapist better apply treatment and diagnoses. Thermal imaging has made huge technological leaps over the last decade with surprising results in terms of the technical specification of the cameras, size and price. Programing applications provide automations of infrared data extraction significantly aiding the time spent in data mining^34^. The user-friendly nature of the technique could find applications in a variety of clinical settings as well as on the pitch since it can provide a direct feedback on the training load of players susceptible to injury. The current study is one of the few that sheds light in the scant use of thermography for the detection of soft tissue injuries. Results provide a novelty in terms of mapping the physiological behavior of temperature after an ankle sprain. Moreover it provides one of the largest studies in terms of sample size that have observed the physiological manifestations of exercise on the ankles as a result of exercise. The only drawback that we have faced experimentally was the poor acquisition of the medial temperature of the non-affected right ankle. Despite the fact that the image was still acquired in a vertical angle the fact that it is different from the rest of the images could have introduced a false recording. Acquisition of data on a different angle compared to the mainstream experimental protocol may jeopardize the interpretations of results leading to false conclusions (see Fig. 3, Day 7). To avoid such experimental limitations the biological movement complications of the patient should be taken into account priory of the design of the experimental protocol. Thus it is crucial for future studies investigating a similar topic to be extremely strict with data harvesting despite the difficulties of the patient in terms of maneuverability. Although the results of our study are promising and in line with previous experimental findings it would be premature to have full confidence on the results since data was obtained on a single sprained ankle. More patients are needed to verify the reproducibility of our results with variable degrees of soft tissue damage.

## Conclusions

The current study was designed to examine the potential of infrared thermal imaging in identifying soft tissue temperature alterations as a result of exercise and as a result of injury. Through the obtained result we have verified that thermal imaging is sensitive enough to delineate variability in temperature that is indicative of both pathology as well as tissue strain. Although exercise and injury may manifest in a similar thermal pattern, an ankle sprain manifests as a more concentrated focal heat patch covering a wider range of tissue in which thermal segmentation extends widely in the surrounding tissue. On the other hand exercise extends beyond the ankle and it is much less dispersed. Segmenting the ankle is a much more successful way in localizing the site of injury rather than relying on the average voxel count and the mean temperature difference to infer pathology. Thermal imaging could be used as an adjacent tool in any environmental setting to assess the magnitude of soft tissue damage independent of common subjective assessment methods such as referred pain and biological movement restriction. Despite that this type of technique is still underdeveloped regarding sports medicine it could be used along side mainstream tools such as MRI to evaluate the level and rate of recovery providing a quantitative assessment of the level of therapy that the athlete receives.
